# Synaptic roles for phosphomannomutase type 2 in a new *Drosophila* congenital disorder of glycosylation disease model

**DOI:** 10.1242/dmm.022939

**Published:** 2016-05-01

**Authors:** William M. Parkinson, Michelle Dookwah, Mary Lynn Dear, Cheryl L. Gatto, Kazuhiro Aoki, Michael Tiemeyer, Kendal Broadie

**Affiliations:** 1 Department of Biological Sciences, Vanderbilt University, Nashville, TN 37235, USA; 2 Department of Biochemistry and Molecular Biology, The University of Georgia, Athens, GA 30602, USA; 3 Kennedy Center for Research on Human Development, Vanderbilt University, Nashville, TN 37235, USA; 4 Complex Carbohydrate Research Center, The University of Georgia, Athens, GA 30602, USA; 5 Department of Cell and Developmental Biology, Vanderbilt University, Nashville, TN 37235, USA

**Keywords:** Synapse, Neurotransmission, Neuromuscular junction, Synaptomatrix, Matrix metalloproteinase, Wnt, Trans-synaptic signaling

## Abstract

Congenital disorders of glycosylation (CDGs) constitute a rapidly growing family of human diseases resulting from heritable mutations in genes driving the production and modification of glycoproteins. The resulting symptomatic hypoglycosylation causes multisystemic defects that include severe neurological impairments, revealing a particularly critical requirement for tightly regulated glycosylation in the nervous system. The most common CDG, CDG-Ia (PMM2-CDG), arises from phosphomannomutase type 2 (PMM2) mutations. Here, we report the generation and characterization of the first *Drosophila* CDG-Ia model. CRISPR-generated *pmm2*-null *Drosophila* mutants display severely disrupted glycosylation and early lethality, whereas RNAi-targeted knockdown of neuronal PMM2 results in a strong shift in the abundance of pauci-mannose glycan, progressive incoordination and later lethality, closely paralleling human CDG-Ia symptoms of shortened lifespan, movement impairments and defective neural development. Analyses of the well-characterized *Drosophila* neuromuscular junction (NMJ) reveal synaptic glycosylation loss accompanied by defects in both structural architecture and functional neurotransmission. NMJ synaptogenesis is driven by intercellular signals that traverse an extracellular synaptomatrix and are co-regulated by glycosylation and matrix metalloproteinases (MMPs). Specifically, trans-synaptic signaling by the Wnt protein Wingless (Wg) depends on the heparan sulfate proteoglycan (HSPG) co-receptor Dally-like protein (Dlp), which is regulated by synaptic MMP activity. Loss of synaptic MMP2, Wg ligand, Dlp co-receptor and downstream trans-synaptic signaling occurs with PMM2 knockdown. Taken together, this *Drosophila* CDG disease model provides a new avenue for the dissection of cellular and molecular mechanisms underlying neurological impairments and is a means by which to discover and test novel therapeutic treatment strategies.

## Abstract (precis)

***Drosophila* Collection:** This work generates a new *Drosophila* congenital disorder of glycosylation model for the most common disease category, caused by phosphomannomutase-2 mutation, and reveals a synaptic mechanism underlying associated neurological impairments.

## INTRODUCTION

Congenital disorders of glycosylation (CDGs), which are caused by mutation of genes encoding glycosylation pathway proteins, are classified into two categories (Freeze et al., 2015): CDG-I disease states include defects in carbohydrate production, lipid-linked oligosaccharide (LLO) formation and attachment of glycan chains to amino acids; CDG-II disease states include defects in the modification and/or maturation of glycan chains after protein attachment. The most common CDG is a CDG-I called CDG-Ia or PMM2-CDG and results from mutations in phosphomannomutase 2 (PMM2), which converts mannose-6-phosphate to mannose-1-phosphate, the obligatory precursor for GDP-mannose production and N-linked glycosylation (Andreotti et al., 2014; Freeze et al., 2014, 2015). Since the first patient in 1980, >100 different mutations in >1000 CDG-Ia patients have been characterized (Freeze et al., 2014; Haeuptle and Hennet, 2009; Jaeken, 2013; Jaeken et al., 1980; Yuste-Checa et al., 2015). CDG-Ia infant mortality is ∼20% in the first year, with affected individuals manifesting subsequent increased susceptibility to organ failure, infection and injury (Grünewald, 2009). Individuals with CDG-Ia present with a spectrum of neurological symptoms (Jaeken, 2013), ranging from severe neurological impairments with early death, to mild defects with slight psychomotor delay (Grünewald, 2009; Marquardt and Denecke, 2003). To date, no effective treatments are available, with the only treatment option being symptom management (Grünewald, 2009; Monin et al., 2014; Stefanits et al., 2014).

CDG-Ia modeling is crucial for molecular and cellular studies. The initial mouse *PMM2* knockout model has been of limited use owing to early embryonic lethality (Thiel et al., 2006). Heteroallelic combination of two PMM2 mutations allows partial enzymatic activity, protracted embryonic survival and has demonstrated potential maternal dietary intervention in treatment (Schneider et al., 2011). Increased lifespan occurred with mannose feeding prenatally and during gestation, reportedly allowing offspring to develop past critical periods of PMM2-dependent glycan requirement (Schneider et al., 2011; Thiel et al., 2006). Unfortunately, these results have thus far not been successfully replicated in patient trials, where postnatal oral and intravenous mannose administration failed to improve serum protein glycosylation levels (Kjaergaard et al., 1998; Mayatepek and Kohlmüller, 1998). A subsequent zebrafish model established via *pmm2* morpholino knockdown revealed increased motor neuron number, altered cranial development, reduced motility and altered glycan profiles (Cline et al., 2012). Most recently, a similar *Xenopus* morpholino knockdown model demonstrated strong reduction in Wnt signaling, revealing a PMM2 requirement in intercellular communication (Himmelreich et al., 2015). These models have been valuable, but have limitations of morpholino-based approaches with inadequate targeting and concerns about the temporal maintenance of knockdown (Schulte-Merker and Stainier, 2014). We therefore set out to develop a *Drosophila* CDG-Ia disease model.

The *Drosophila* genome encodes >70% of human disease genes (Reiter et al., 2001), including most linked to glycan-related disorders (Dani et al., 2012, 2014). The human N-linked glycome is more expansive than that of *Drosophila*, with higher levels of complex and hybrid branched forms (Katoh and Tiemeyer, 2013), but the N-glycosylation pathway is very highly conserved, with well-mapped glycan profiles and the *Drosophila* genetic toolkit allowing sophisticated manipulation (Altmann et al., 2001; Sarkar et al., 2006). For neurological impairments (Freeze et al., 2015; Jaeken, 2013; Martin, 2003), *Drosophila* provides a host of anatomical, electrophysiological and behavioral assays to study neural development and function (Dani et al., 2014; Gatto and Broadie, 2011; Jumbo-Lucioni et al., 2014; Parkinson et al., 2013). In particular, our glycomic RNA interference (RNAi) screen uncovered a common role for glycosylation in restricting the structure and function of the neuromuscular junction (NMJ) synapse (Dani et al., 2012). Subsequent work on screen hits, including heparan sulfate proteoglycan (HSPG) sulfotransferase (*hs6st*), sulfatase (*sulf1*), UDP-GlcNAc:α-3-D-mannoside-β1,2-N-acetylglucosaminyl-transferase I (*mgat1*), α-N-acetylgalactosaminyltransferase (*pgant*), galactose-1-phosphate uridyl-transferase (*galt*) and related pathways (Dani et al., 2012, 2014; Jumbo-Lucioni et al., 2014; Parkinson et al., 2013), has shown disruption of trans-synaptic signaling to be a common root cause of the compromised NMJ synaptogenesis underlying movement phenotypes.

In this study, we generate a *Drosophila* CDG-Ia disease model. We show that *Drosophila pmm2* is highly conserved, and we manipulate gene function by making mutants with clustered regularly-interspaced short palindromic repeat (CRISPR)/Cas9 genome editing and tissue-targeted transgenic RNAi. As in humans with CDG-Ia, *Drosophila pmm2* loss-of-function (LOF) mutants exhibit reduced lifespan and psychomotor retardation proportional to the degree of PMM2 reduction. We show striking impacts on the N-linked glycome: globally in null mutants, neurally in tissue-targeted RNAi knockdown and locally at the NMJ synapse. At the NMJ, targeted pre- and post-synaptic PMM2 knockdown reveals architectural overelaboration, and concurrent reduction on both sides of the synapse strongly increases neurotransmission strength. PMM2 loss strongly impairs the synaptic matrix metalloproteinase (MMP) pathway regulating the HSPG co-receptor Dally-like protein (Dlp) to modulate Wnt Wingless (Wg) trans-synaptic signaling (Dani et al., 2012; Dear et al., 2016; Friedman et al., 2013). Consistently, PMM2 knockdown reduces Wg, Dlp and the Frizzled nuclear import (FNI) pathway. These results suggest that a PMM2-dependent extracellular proteinase mechanism modulates Wnt signaling during NMJ synaptogenesis, which underlies coordinated movement and maintained viability. This new *Drosophila* model should lead to novel therapeutic treatments for CDG-Ia.

## RESULTS

### Degree of *Drosophila PMM2* loss determines lifespan duration

**Fig. 1.**
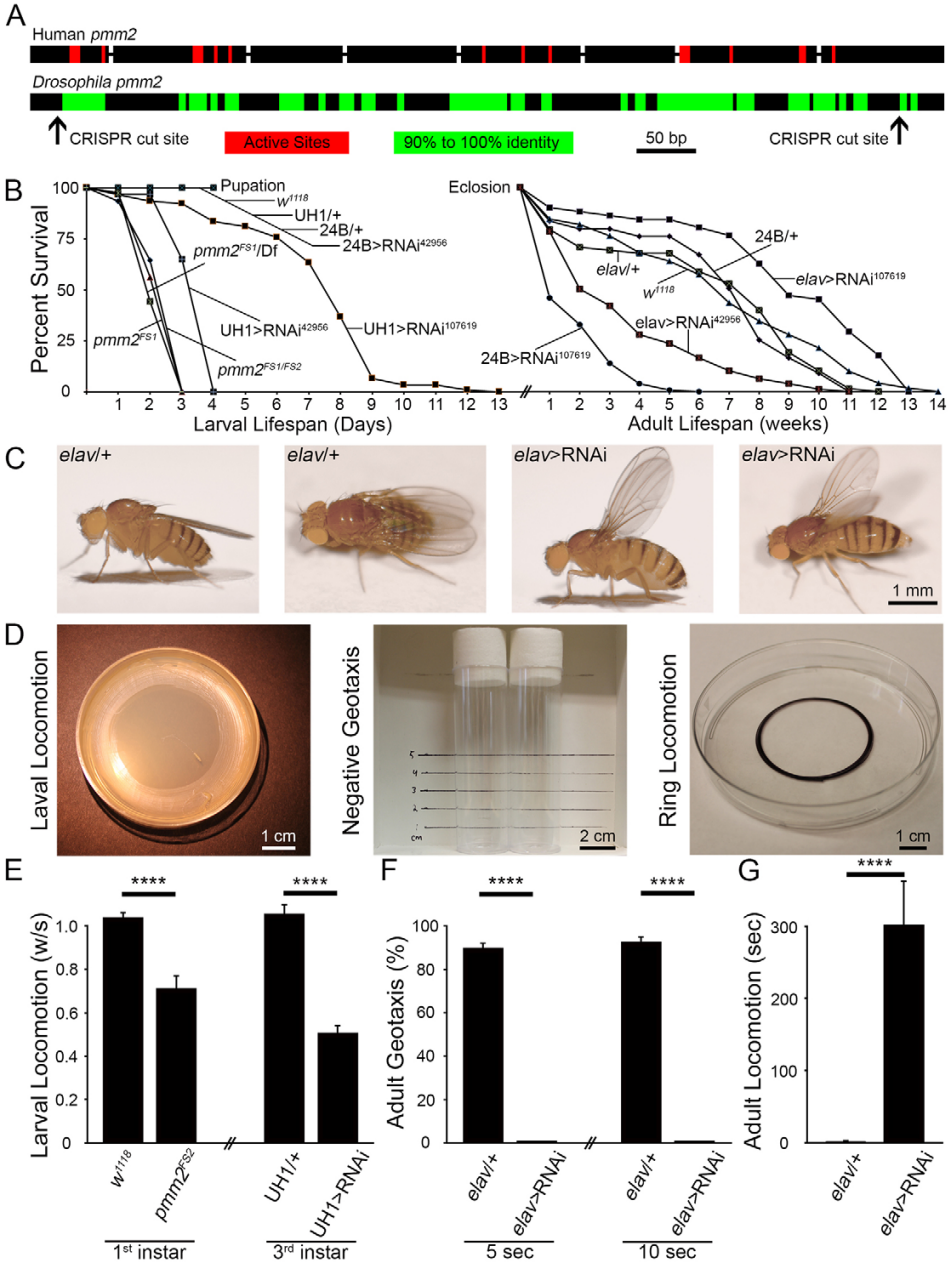
***Drosophila* PMM2 levels determine lifespan and coordinated movement ability.** (A) Comparison of human and *Drosophila pmm2* genes. Human *PMM2* contains eight exons (black boxes) compared to the single reading frame in *Drosophila*. Highly conserved active sites (red) and other regions of high identity (90-100%, green) are depicted. CRISPR-generated null mutants were made at both 5′ and 3′ ends of *pmm2* (arrows). (B) Larval lifespan of null mutants (*pmm2^FS1^*, *pmm2^FS1^*/Df and heteroallelic *pmm2^FS1/FS2^*) and two driven RNAi lines (RNAi^42956^ and RNAi^107619^) compared to controls (left). Adult lifespan with targeted neural (*elav*-Gal4) and muscle (24B-Gal4) *pmm2* RNAi compared to controls (right). (C) Examples of wing posture with neural *pmm2* RNAi. (D) Behavioral assays: larval locomotion on apple juice plates towards edge yeast attractant (left); adult negative geotaxis climbing to 2 cm height (middle), and adult horizontal locomotion for flies with wings removed exiting a 4 cm ring (right). (E) Normalized quantification of 1st (left) and 3rd instar (right) larval locomotion (peristaltic waves/second) in genotypes shown. (F) Quantification of adult negative geotaxis as percent animals climbing to 2 cm height at 5 (left) and 10 (right) seconds. (G) Quantification of adult horizontal locomotion as time to exit a 4 cm ring. Significance: *P*<0.0001 (****). Sample sizes: *n*≥17 animals/genotype.

Human *PMM2* contains eight exons, compared to the *Drosophila* CG10688 *pmm2* single reading frame (Fig. 1A), making genetic manipulation and expression studies more amenable. Simplified site-directed CRISPR mutagenesis targeted at one exon reduces the possibility of truncated exons maintaining partial function. Human PMM2 contains 12 active sites that coordinately bind the substrate, mannose-6-phosphate, and convert it to mannose-1-phosphate (Andreotti et al., 2014; Silvaggi et al., 2006). *Drosophila* PMM2 shows 100% conservation of these 12 active sites (Fig. 1A, red) and identifies numerous additional regions of high (90-100%) conservation (Fig. 1A, green). Overall, *Drosophila* PMM2 displays 56% amino acid identity with human PMM2 (Fig. 1A). To characterize the role of PMM2, we first made null mutants using CRISPR/Cas9 genome editing directed at both the 5′ and 3′ ends of *Drosophila pmm2* (Fig. 1A; Gratz et al., 2013). A total of 15 mutations were produced, all verified with direct sequencing: eight frameshifts, four insertions, two deletions and one missense mutation. Two *pmm2-*null frameshift mutations were selected for subsequent behavioral, functional and molecular studies, hereafter referred to as *pmm2^FS1^* and *pmm2^FS2^*.

Two independent UAS-RNAi lines (Bloomington *Drosophila* Stock Center #42956 and Vienna *Drosophila* Resource Center v107619) were used to differentially reduce *pmm2* levels. Quantitative PCR (qRT-PCR) with ubiquitous (UH1-Gal4) expression shows that both RNAi lines knock down *pmm2* transcripts by >77%, a significant (*P*<0.001) reduction compared to UH1-Gal4/+ transgenic controls. Of the two lines, 42956 is significantly more effective than 107619 in reducing *pmm2* levels (values normalized to ribosomal protein L32: UH1>RNAi^107619^, 1.57±0.10; UH1>RNAi^42956^, 1.27±0.10; *n*=15, *P*=0.016) compared to control (UH1-Gal4/+, 7.00±0.79, *n*=15). Moreover, at 50% lethality of ubiquitous *pmm2* knockdown (42956) (Fig. 1B), *pmm2* levels in UH1-Gal4/+ controls showed a sharp increase (1st instar, 1.40±0.15, *n*=8; 2nd instar 7.00±0.79, *n*=15), which is paralleled by the surviving ubiquitous *pmm2* knockdown (107619), permitting higher levels of maintained *pmm2* expression. The strong (42956) and weak (107619) PMM2 knockdown phenotypes are likely the result of the significant differences in transcript levels.

Null *pmm2* mutants, heteroallelic combinations and over-deficiency mutants all show identical early larval lethality during the late 1st to early 2nd instar transition (Fig. 1B). Half-time survival (HTS) is similar [e.g. *pmm2^FS1^* HTS 51 h post-hatching (hph), *n*=73; *pmm2^FS1^*/Df HTS 46 hph, *n*=79; *pmm2^FS2^*/Df HTS 51 hph, *n*=84; *pmm2^FS1^*/*pmm2^FS2^* HTS 52 hph, *n*=75]. Ubiquitous *pmm2* knockdown (42956) results in a lifespan that is only marginally longer than *pmm2* nulls (HTS: 68 hph; *n*=63), whereas the weaker ubiquitous *pmm2* knockdown (107619) allows survival to late larval stages (HTS: 168 hph; *n*=90) with some pupation (mean 192 hph; Fig. 1B). UH1/+ controls pupate at 96 hph, showing that ubiquitous *pmm2* knockdown (107619) exhibits severe developmental delay. Muscle-specific 24B-Gal4 with the stronger RNAi^42956^ produces 100% pupal lethality, whereas weaker 24B>RNAi^107619^ allows survival but with reduced adult lifespan (HTS: 10 days; *n*=159) compared to control (24B-Gal4/+, HTS: 38 days; *n*=79; Fig. 1B). Targeted neural *elav*-Gal4 with RNAi^42956^ exhibits similarly reduced adult lifespan (HTS: 17 days; *n*=92), which is conversely extended with weak *elav*>RNAi^107619^ (HTS: 65 days; *n*=51) compared to control (*elav*-Gal4/+, HTS: 39 days; *n*=68; Fig. 1B). This suggests a delicate PMM2 balance, with strong neural loss being detrimental but moderate neural loss extending lifespan. Owing to these lethality constraints, hereafter weaker RNAi^107619^ is used for ubiquitous knockdown and stronger RNAi^42956^ is used for tissue-targeted neural and muscle knockdown.

### Neuronal PMM2 maintains normal posture and coordinated movement

Neurological movement symptoms in individuals with CDG-Ia range from slight gait ataxia to severe cerebellar ataxia; most children are unable to walk unassisted, and most adults are wheel-chair bound (Barone et al., 2014, 2015; Marquardt and Denecke, 2003; Monin et al., 2014). Loss of *Drosophila* PMM2 results in similarly severe postural and movement phenotypes, largely due to nervous system involvement. Targeted neural *elav*-Gal4 knockdown results in a highly penetrant ‘held-out wings’ posture (Fig. 1C), which is associated with defects in flight-muscle control of wing positioning (Müller et al., 2010; Zaffran et al., 1997). Neural *pmm2* knockdown adults have severe ataxia, with profound incoordination, inability to walk in a directed fashion and complete inability to fly. Weak muscle-specific PMM2 knockdown does not yield ataxia. However, strong muscle-specific knockdown allows pupae to develop fully, but not a single pharate adult properly ecloses. A few animals partially eclose before dying, and animals mechanically freed from pupal cases do not move or survive. Based on these severe qualitative movement defects, we conducted a range of quantitative analyses (Fig. 1D).

Larval locomotion requires a CNS pattern generator that drives coordinated stimulation of segmental nerves to drive NMJ transmission, evoking coordinated muscle contraction (Gjorgjieva et al., 2013; Kohsaka et al., 2014; Nichols et al., 2012; Sokolowski, 1980). Locomotion was assayed on apple juice agar plates, monitoring movement from the barren center to a yeast reward located along the rim (Fig. 1D, left). At 24 hph, *pmm2*-null 1st instar mutants display a significant reduction in coordinated movement, as measured in peristaltic waves/second (*pmm2^FS2^*, 0.71±0.06 w/s; *n*=17), compared to the genetic control (*w^1118^*, 1.04±0.02 w/s; *n*=17; *P*<0.0001; Fig. 1E). Both lifespan and movement data show that ubiquitous *pmm2* knockdown (42956) phenocopies the *pmm2* genetic nulls. Similarly, 3rd instar ubiquitous *pmm2* knockdown (107619) larvae show a comparable reduction in larval locomotion (0.51±0.04 w/s; *n*=20) compared to controls (UH1-Gal4/+, 1.06±0.04 w/s; *n*=20, *P*<0.0001; Fig. 1E). Thus, global PMM2 loss dramatically impairs the coordinated movement needed to maintain viability.

Neural-specific PMM2 involvement was next tested in adults, using a range of established assays (Nichols et al., 2012). Negative geotaxis assays the animals’ ability to climb vertically (2 cm) from the bottom of a vial (Fig. 1D, middle). Neurally targeted *elav*-Gal4>RNAi^42956^ prevents this simple task, whereas nearly all control animals display the necessary coordinated locomotion at 5 s (*elav*-Gal4/+, 90±2.1%, *n*=50) and 10 s (*elav*-Gal4/+, 93±2.4%, *n*=50; Fig. 1F). Not a single *pmm2* RNAi animal performed the task (*n*=40, *P*<0.0001). A less demanding horizontal locomotion assay measures the time required to escape a 4 cm diameter circle (Fig. 1D, right). Neurally targeted *elav*-Gal4>RNAi^42956^ results in a 150-fold delay (302.0±60.02 s; *n*=15) compared to controls (*elav*-Gal4/+, 1.93±0.32 s; *n*=15; *P*<0.0001; Fig. 1G). Mutants exit the ring by uncoordinated flailing/kicking, with only a few animals crossing the circle in a directed, purposeful manner. These results show a strong PMM2 role for coordinated movement, similar to CDG-Ia symptoms in humans, with a striking PMM2 impact on neurons.

### PMM2 loss suppresses N-glycosylation and enhances glycan turnover

**Fig. 2.**
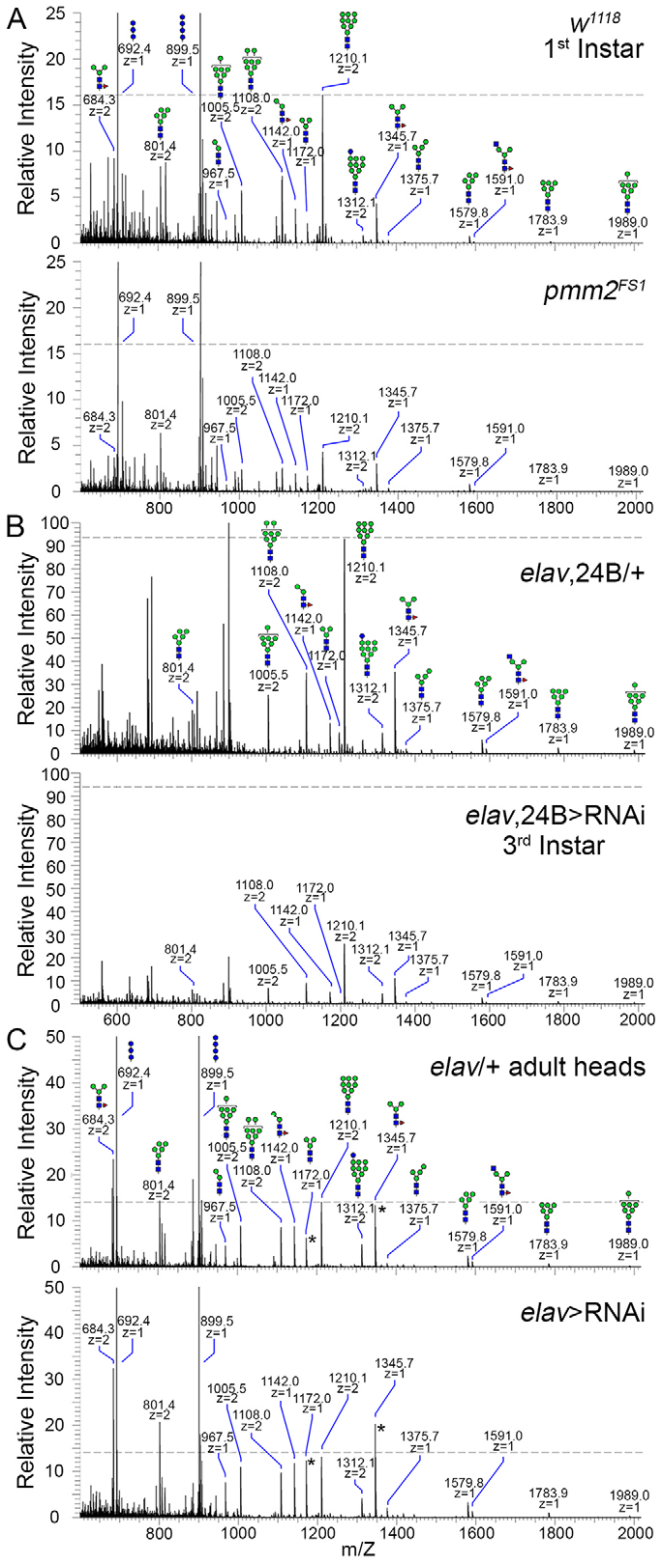
**PMM2 loss dramatically alters N-linked glycoprotein glycosylation.** Full mass spectrometry (MS) spectra for permethylated glycans harvested from (A) age-matched *w^1118^* genetic control (top) and *pmm2^FS1^*-null (bottom) 1st instars, (B) *elav-*Gal4*,*24B-Gal4/+ transgenic control (top) and *elav-*Gal4*,*24B-Gal4>RNAi^42956^ (bottom) 3rd instars, and (C) *elav*-Gal4/+ transgenic control (top) and *elav*-Gal4>RNAi^42956^ (bottom) adult heads. Dashed lines indicate the relative abundance of Man_9_GlcNAc_2_ (m/z=1210.1, doubly-charged) in each control for comparison with mutants across genotypes. In all panels, mass peaks at m/z=692.4 and 899.5 are Hex_3_ and Hex_4_ glycans spiked into samples as external calibration standards. The cartoon representations of glycan structures are shown in accordance with glycomics community conventions (Varki and Sharon, 2009). Asterisks define peaks to highlight selected changes.

To assess N-linked glycoprotein glycosylation levels correlating with movement defects in *pmm2*-null mutants and upon neurally targeted RNAi knockdown, we next assayed glycome composition. Humans with CDG-Ia display altered glycosylation status, including reduced concanavalin A (ConA) binding but increased fucose-decorated glycoproteins (Van Dijk et al., 2001). In *Drosophila*, at all stages of development and in all tissues, the major N-linked glycoprotein constituent, glycans, include high-mannose and pauci-mannose (≥three mannose residues) classes, whereas human glycosylation typically results in more branched and complexly decorated proteins (ten Hagen et al., 2009; Katoh and Tiemeyer, 2013). Complex hybrid and branched N-glycans are present in *Drosophila*, but at much lower relative abundances (Aoki et al., 2007). Here, *Drosophila* N-linked glycans were analyzed by mass spectrometry (MS) throughout the larval body in *pmm2*-null mutants and with combined neuronal (*elav*-Gal4) and muscle (24B-Gal4) *pmm2* RNAi, and in adult heads only with neurally targeted *pmm2* RNAi (Fig. 2).

N-linked glycans were released from *pmm2*-null (*pmm2^FS1^*) compared to genetic background control (*w^1118^*) from equal amounts of protein. Proteins analyzed by MS were dramatically reduced for all major classes (high-mannose, pauci-mannose and complex; Fig. 2A). For example, the relative abundance of Man_9_GlcNAc_2_ [mass-to-charge ratio (m/z)=1210.1, doubly-charged], a dominant structure in the glycan profile, was reduced fourfold in *pmm2*-null mutants (Fig. 2A; dashed line) compared with controls. All other detected N-glycans exhibited comparable reductions, consistent with a global suppression of glycoprotein glycosylation. A similar global reduction occurred in 3rd instar larva using paired neural and muscle Gal4s to drive *pmm2* RNAi (*elav*,24B>RNAi^42956^; Fig. 2B). Interestingly, neurally targeted *pmm2* RNAi in the adult head did not produce the same phenotype. N-linked glycan quantities were relatively comparable for transgenic control (*elav*-Gal4/+) and this targeted *pmm2* knockdown (*elav*-Gal4>RNAi^42956^; Fig. 2C). Major complex N-linked glycans (e.g. NM_3_N_2_F, m/z=1591.0, singly-charged) were not noticeably changed in abundance. However, pauci-mannose glycans displayed clearly increased abundance. For example, the core-fucosylated trimannosyl glycan M_3_N_2_F (m/z=1345.7, singly-charged) was increased in relative abundance, as was non-fucosylated M_3_N_2_ (m/z=1172.0, singly charged), with *pmm2* knockdown (Fig. 2C).

**Fig. 3.**
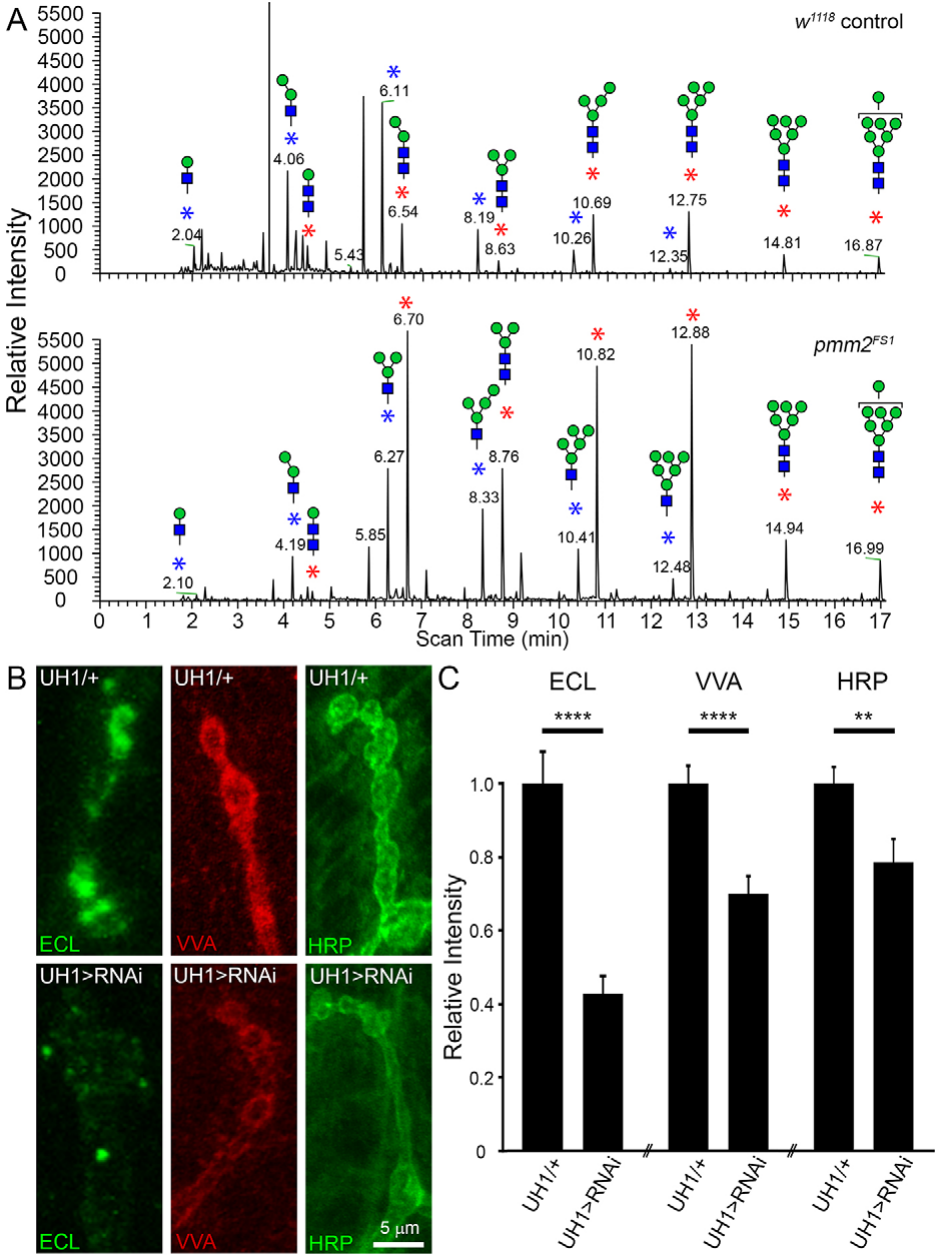
**PMM2 loss increases FOS and reduces NMJ N-linked glycoprotein glycosylation.** (A) Free oligosaccharides (FOS) from *w^1118^* genetic control and *pmm2^FS1^*-null 1st instar larvae. FOS are detected as products of sequential PNGase and endo-N-acetylglucosaminidase digestion (N1, blue asterisks), or as glycans with two reducing terminal GlcNAc residues (N2, red asterisks). Total ion mapping chromatograms were filtered for loss of non-reducing terminal HexNAc residues to show the two FOS classes. (B) Representative images of *Erythrina cristagalli* (ECL, green; left), *Vicia villosa* (VVA, red; middle) and horseradish peroxidase (HRP, green; right) NMJ labeling in *pmm2* knockdowns (bottom) compared to transgenic controls (top). (C) Quantification of fluorescent labeling intensity in UH1-Gal4/+ transgenic control versus UH1-Gal4>RNAi^107619^. Significance: *P*≤0.01 (**), *P*≤0.0001 (****). Sample sizes: *n*≥24 NMJs/12 animals per genotype.

Loss of N-linked glycoprotein glycosylation in *pmm2^FS1^* mutants implies decreased efficiency in glycosylation initiation by the oligosaccharyltransferase complex (OST) in the endoplasmic reticulum (ER), and/or increased ER-associated protein degradation (ERAD)-mediated protein deglycosylation within the cytoplasm (Gao et al., 2011). Either mechanism should be reflected by increased abundance of free oligosaccharide (FOS), branched oligosaccharides unbound to lipid or protein, either owing to endogenous peptide-N-glycosidase F (PNGase) release or via lipid-linked precursor hydrolysis by the OST (Cline et al., 2012; Gao et al., 2011). Consistently, FOS abundance was strikingly increased in *pmm2^FS1^* mutants (Fig. 3A). FOS abundance was detected as products of PNGase and endo-N-acetylglucosaminidase digestion (N1; Fig. 3A, blue asterisks) or glycans possessing two reducing terminal GlcNAc residues (N2; Fig. 3A, red asterisks), with total ion mapping chromatograms filtered for loss of non-reducing terminal HexNAc residues. Both FOS classes were increased in *pmm2* nulls compared to controls (Fig. 3A). Thus, glycomics and FOS assays show striking reduction in mature glycan abundance globally, and an altered glycomic repertoire in the nervous system, with PMM2 loss. To test for a cellular role, we next moved to the well-characterized NMJ that drives movement.

### PMM2 maintains the normal glycan environment in the NMJ synaptomatrix

Glycan-binding lectin probes have long been used to chart the cellular carbohydrate landscape both at mammalian and *Drosophila* synapses (Jumbo-Lucioni et al., 2014; Parkinson et al., 2013; Schneider et al., 2011). To assay the composition of the heavily glycosylated larval NMJ synaptomatrix, we utilized a panel of lectins, including: *Erythrina cristagalli* (ECL) to label D-galactose glycans; *Vicia villosa* (VVA) to label N-acetylgalactosamine glycans; and anti-horseradish peroxidase (HRP) to label alpha1-3-linked fucose moieties (Jumbo-Lucioni et al., 2014; Kurosaka et al., 1991). Both ECL and VVA revealed strong reductions in ubiquitous *pmm2* knockdown (107619) compared to the UH1-Gal4/+ controls (Fig. 3B). Quantification revealed >50% ECL reduction (UH1-Gal4>RNAi^107619^, 0.43±0.05; UH1-Gal4/+, 1.00±0.08; *n*=24; *P*<0.0001) and >30% VVA reduction (UH1-Gal4>RNAi^107619^, 0.69±0.05; UH1-Gal4/+, 1.00±0.05; *n*=24; *P*<0.0001; Fig. 3C). HRP is reduced by ≥20% (UH1-Gal4>RNAi^107619^, 0.79±0.06; UH1-Gal4/+, 1.00±0.06; *n*=24; *P*≤0.01; Fig. 3C). Dual neural and muscle knockdown *elav*-Gal4,24B-Gal4 RNAi also exhibited ≥20% HRP loss (*elav*-Gal4,24B>RNAi^42956^, 0.79±0.06; *elav*-Gal4,24B/+, 1.0±0.04; *n*≥26, *P*<0.008). These results indicate a robustly altered glycan composition within the NMJ synaptomatrix, which earlier studies have linked to defects in NMJ structural and functional development.

### PMM2 restricts NMJ structural growth and synaptic bouton differentiation

**Fig. 4.**
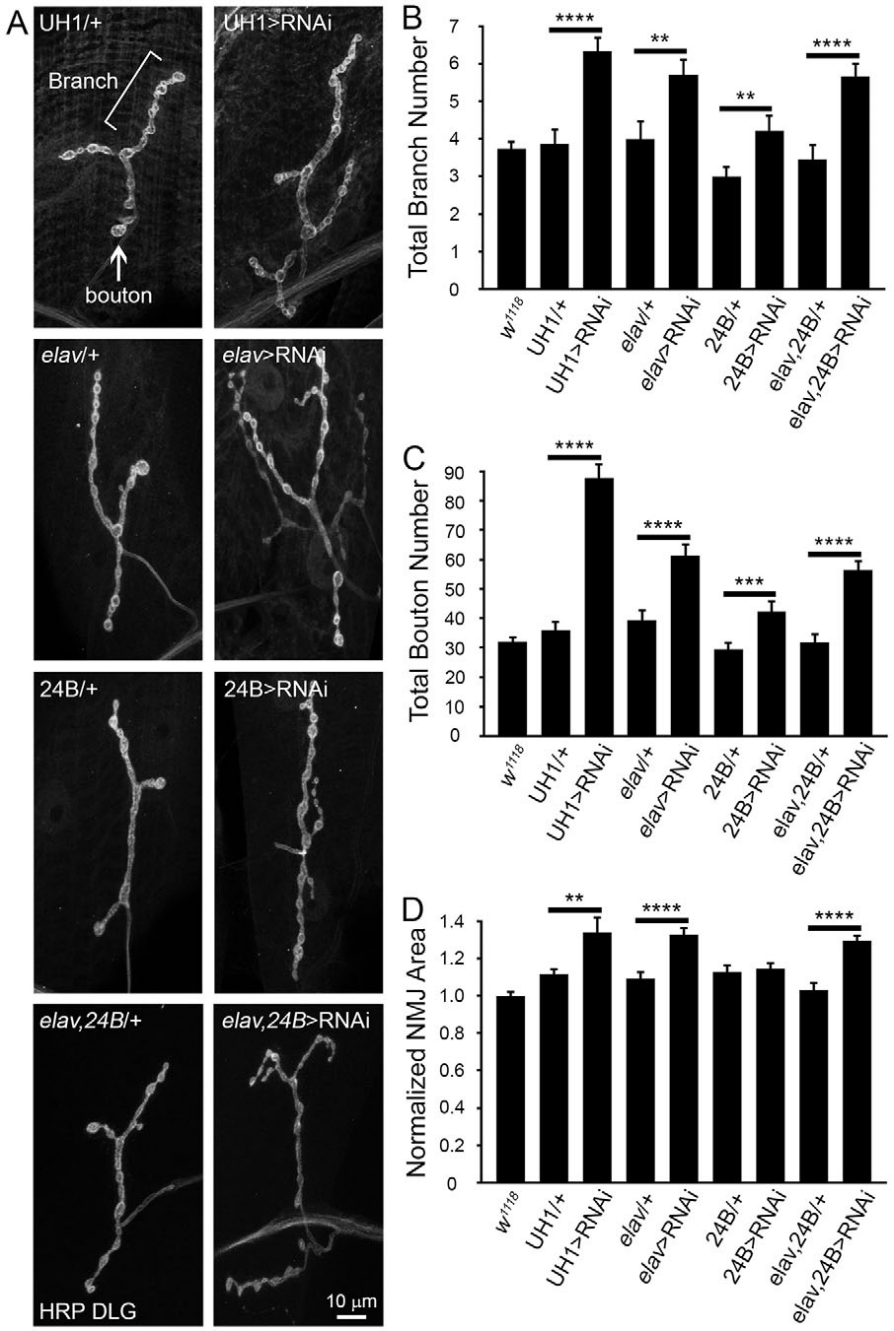
**PMM2 loss results in striking NMJ synaptic structural overgrowth.** (A) Representative wandering 3rd instar muscle 4 NMJ images of ubiquitous (UH1, top), neural (*elav*, second), muscle (24B, third) and double (*elav*, 24B, bottom) driver controls (left column), compared to RNAi-mediated *pmm2* knockdown (right). NMJs are co-labeled for pre-synaptic HRP and post-synaptic DLG, merged and shown in black-and-white to best illustrate structure. Quantification of NMJ branch number (B), bouton number (C) and terminal area (D) in the above eight genotypes compared to *w^1118^* (nine genotypes total). Significance: *P*≤0.01 (**), *P*≤0.001 (***), *P*≤0.0001 (****). Sample sizes: *n*≥22 NMJs/12 animals for each genotype.

Loss of synaptomatrix glycosylation has been shown to result in elevated structural elaboration of the *Drosophila* NMJ, as evidenced by increased branching and excess synaptic boutons (Jumbo-Lucioni et al., 2014; Parkinson et al., 2013). To examine NMJ architecture, we characterized wandering 3rd instar muscle 4 NMJs labeled for HRP and Discs large (DLG) to illuminate pre- and post-synaptic compartments, respectively (Fig. 4A). Synaptic branches (defined as HRP-positive processes with >two boutons), boutons (defined as DLG-positive synaptic varicosities >1 µm in diameter) and NMJ terminal area (based on DLG-positive labeling) were all quantified (Fig. 4B-D). Cell-specific roles were tested with neuronal (*elav*)- and muscle (24B)-targeted *Gal4* drivers in comparison to ubiquitous (UH1-Gal4) *pmm2* knockdown. In all cases, loss of PMM2 resulted in clear NMJ overelaboration, with more branches and supernumerary type Is/Ib synaptic boutons, with ubiquitous RNAi resulting in the greatest level of unrestrained NMJ overgrowth (Fig. 4).

PMM2 loss increases NMJ branching, owing to both pre- and post-synaptic roles (Fig. 4A). Ubiquitous *pmm2* RNAi increases branch number ∼twofold (UH1-Gal4>RNAi^107619^, 6.3±0.36 branches, *n*=24) compared to controls (*w^1118^*, 3.7±0.19 branches, *n*=88; UH1-Gal4/+, 3.9±0.38 branches, *n*=22; *P*<0.0001; Fig. 4B). Likewise, neural (*elav*) and muscle (24B) knockdown more weakly increase terminal branch number (*elav*, 5.7±0.40 branches; 24B, 4.2±0.39 branches; *elav*,24B, 5.7±0.33 branches) compared to transgenic controls (*elav-Gal4*/+, 4.0±0.47 branches; 24B-Gal4/+, 3.0±0.24 branches; *elav*,24B-Gal4/+, 3.5±0.37 branches; *P*<0.01, *P*<0.01 and *P*<0.0001, respectively; Fig. 4B). Consistently, bouton number is elevated >twofold with ubiquitous PMM2 removal (UH1-Gal4>RNAi^107619^, 88±3.9 boutons, *n*=24) relative to controls (*w^1118^*, 32±1.4 boutons, *n*=88; UH1-Gal4/+, 36±2.4 boutons, *n*=22; *P*<0.0001; Fig. 4C). Bouton number is also elevated with neural and muscle knockdown (*elav*, 61±3.8 boutons; 24B, 42±3.0 boutons; *elav*,24B, 56.5±2.9 boutons) compared to controls (*elav-Gal4*/+, 39±3.3 boutons; 24B-Gal4/+, 30±2.0 boutons; *elav*,24B/+, 31.8±2.7 boutons; *P*<0.0001, *P*<0.001 and *P*<0.0001, respectively; Fig. 4C). Finally, analyses of synaptic terminal area showed increased size with ubiquitous, neuronal and co-neural/muscle PMM2 loss (*P*<0.01, *P*<0.0001 and *P*<0.0001, respectively), but with no significant change upon muscle-specific PMM2 removal (Fig. 4D).

**Fig. 5.**
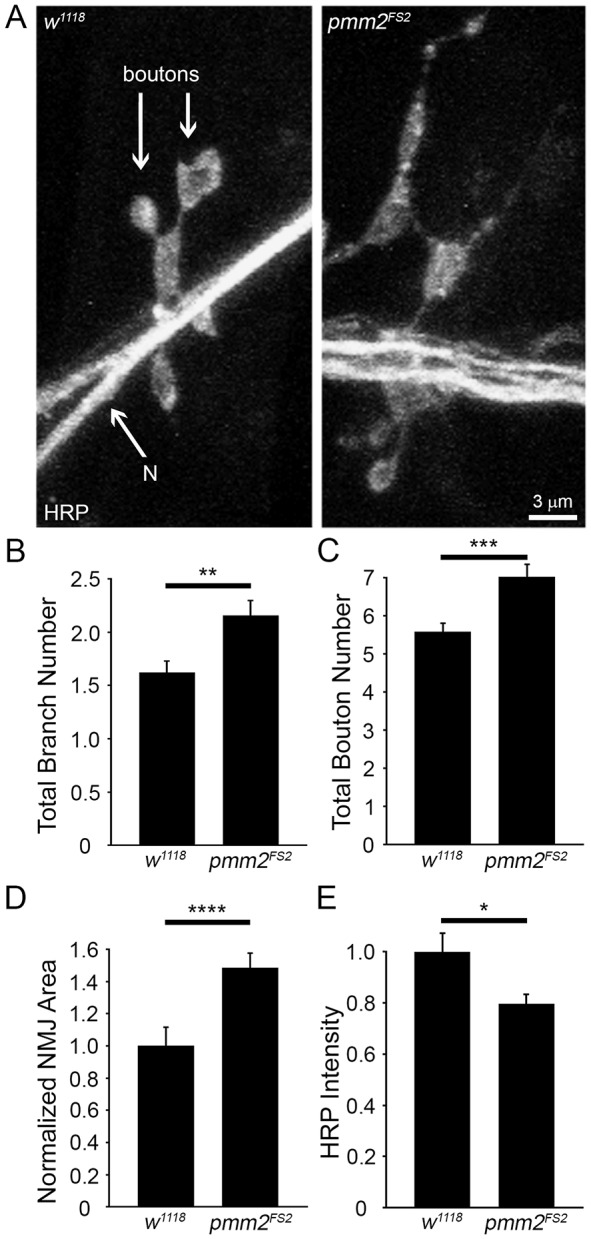
**Early lethal *pmm2*-null mutants exhibit NMJ overgrowth and loss of N-glycans.** (A) Representative 1st instar muscle 4 NMJ images from genetic background control (*w^1118^*, left) and *pmm2*-null mutant (*pmm2^FS2^*, right). NMJs are labeled for the pre-synaptic anti-HRP. Quantification of synaptic branch number (B), bouton number (C), synaptic terminal area (D) and HRP fluorescence labeling intensity (E). Significance: *P*≤0.05 (*), *P*≤0.01 (**), *P*≤0.001 (***) and *P*<0.0001 (****). Sample sizes: *n*≥30 NMJs/13 animals per genotype. N, nerve.

Synaptic over-elaboration defects occurred across the neuromusculature. Compared to the above lateral muscle 4 defects (Fig. 4), ventral muscles 6/7 exhibited comparable NMJ phenotypes. For example, the synaptic area in *elav*-Gal4,24B-Gal4/+ transgenic controls (380.82 µm^2^) was dramatically expanded in neural and muscle combined-knockdown *elav*-Gal4,24B-Gal4<RNAi^42956^ flies (526.82 µm^2^), a highly significant (*P*>0.0001) increase. Early lethal *pmm2* genetic null mutants also exhibited similarly increased NMJ growth and structural elaboration in the 1st instar prior to developmental arrest (Fig. 5). Using the structural parameters as above, *pmm2* nulls manifested increased synaptic branch number (*w^1118^*, 1.6±0.11 branches; *pmm2^FS2^*, 2.2±0.14 branches; *P*=0.007; Fig. 5B), more bouton formation (*w^1118^*, 5.6±0.21 boutons; *pmm2^FS2^*, 7.0±0.32 boutons; *P*=0.0006; Fig. 5C) and dramatically increased synaptic area (*w^1118^*, 1.0±0.07; *pmm2^FS2^*, 1.5±0.05; *P*=0.0001; Fig. 5D). Consistently, *pmm2-*null mutants exhibited a loss of synaptic N-linked glycosylation, including ≥20% decrease in HRP glycan labeling (*w^1118^*, 1.0±0.08; *pmm2^FS2^*, 0.80±0.04; *n*≥30, *P*≤0.05; Fig. 5E). To determine how NMJ overgrowth compares to synaptic function, we next turned to electrophysiological studies.

### Coupled pre- and post-synaptic PMM2 function limits NMJ transmission strength

**Fig. 6.**
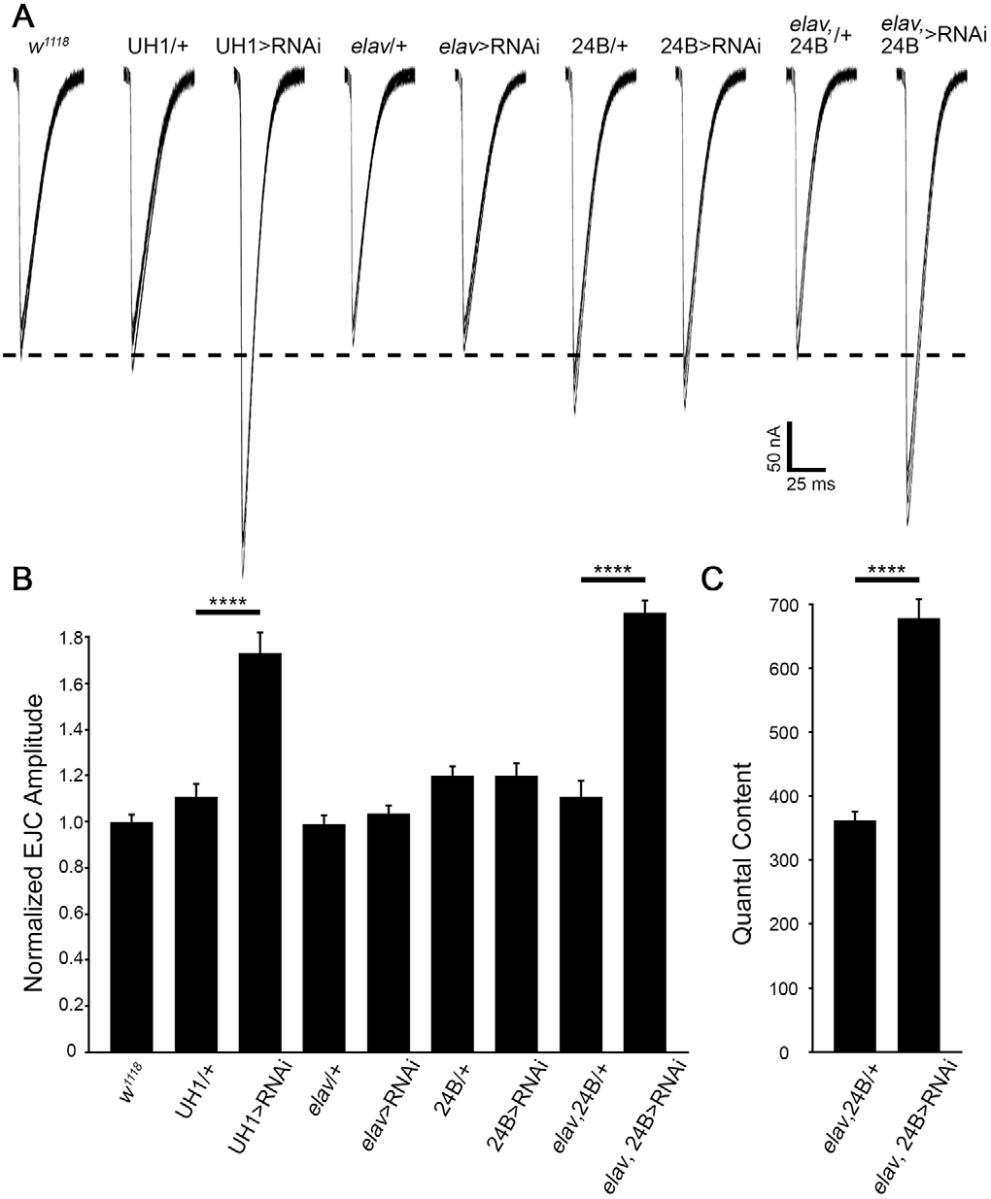
**Coupled pre- and post-synaptic PMM2 removal increases neurotransmission.** (A) Representative two-electrode voltage-clamp (TEVC) recordings of nerve-stimulation-evoked excitatory junctional currents (EJCs) from wandering 3rd instar muscle 6. Superimposed traces are shown in response to nerve stimulation at 1.0 mM Ca^2+^ comparing UH1-Gal4>RNAi^107619^, *elav*-Gal4>RNAi^42956^, 24B-Gal4>RNAi^42956^ and *elav*-Gal4, 24B-Gal4>RNAi^42956^ to *w^1118^* and *Gal4*-driver-alone controls. Quantification of peak EJC amplitude normalized to *w^1118^* (B) and neurotransmission quantal content (C). Significance: *P*≤0.0001 (****). Sample sizes: *n*≥16 NMJs/8 animals per genotype.

Glycosylation has been shown to play key roles in NMJ functional differentiation and the determination of neurotransmission strength (Dani et al., 2012, 2014; Parkinson et al., 2013). The severely impaired coordinated locomotion and alterations in NMJ structure similarly suggest that PMM2 has roles in synaptic function. We tested neurotransmission in the two-electrode voltage-clamp (TEVC) recording configuration by stimulating the motor nerve with a glass suction electrode and measuring the evoked excitatory junctional current (EJC) from the voltage-clamped muscle (Parkinson et al., 2013). To compare EJC transmission properties, ten consecutive stimulation recordings were made at 0.2 Hz, and then averaged to calculate the mean peak transmission amplitude. Cell-specific roles in functional differentiation were tested with targeted neuronal (*elav*) and muscle (24B) *Gal4* drivers, alone and in combination, in comparison to ubiquitous (UH1-Gal4) *pmm2* RNAi knockdown (Fig. 6).

PMM2 loss dramatically increased neurotransmission strength, owing to an unusual coupled role in both pre- and post-synaptic cells (Fig. 6A). Sample recordings show that all transgenic controls and the genetic background control (*w^1118^*) had comparable EJC properties, and only ubiquitous and combined pre- and post-synaptic PMM2 knockdown strongly and equally increased transmission amplitude (Fig. 6A). Ubiquitous RNAi increases peak EJC values by ∼75% [UH1-Gal4>RNAi^107619^, 1.73±0.09 (450.2±23.72 nA), *n*=21; *w^1118^*, 1.00±0.03 (253.68±8.26 nA), *n*=44; UH1-Gal4/+, 1.11±0.06 (288.57±14.39 nA), *n*=21; *P*<0.0001; Fig. 6B]. Strong PMM2 knockdown in either neuron [*elav-*Gal4>RNAi^42956^, 1.03±0.04 (301.8±12.66 nA), *n*=16] or muscle [24B-Gal4>RNAi^42956^, 1.20±0.05 (320.7±13.41 nA), *n*=18] had no significant impact on amplitude compared to controls [*elav*-Gal4/+, 0.99±0.04 (289.9±12.09 nA), *n*=17; 24B-Gal4/+, 1.20±0.04 (302.4±10.90 nA), *n*=16; Fig. 6B]. However, coincident *pmm2* RNAi driven in both the pre-synaptic neuron and post-synaptic muscle again showed significantly (*P*<0.0001) increased EJCs [*elav*-Gal4,24B-Gal4>RNAi^42956^, 1.91±0.05 (397.61±11.58 nA), *n*=23] compared to the dual-driver control alone [*elav*-Gal4,24B-Gal4/+, 1.11±0.07 (231.0±14.56 nA), *n*=23; Fig. 6B]. We next recorded miniature evoked junction currents (mEJCs) in neural and muscle combined-knockdown *elav*-Gal4,24B-Gal4>RNAi^42956^ larvae but did not detect any significant change in either frequency (*elav*-Gal4,24B-Gal4/+, 1.80±0.11 Hz; *elav*-Gal4,24B-Gal4/42956, 1.93±0.14 Hz; n.s., *n*=20) or amplitude (*elav*-Gal4,24B-Gal4/+, 0.66±0.02 nA; *elav*-Gal4,24B-Gal4/42956, 0.61±0.03 nA; n.s., *n*=20) compared with control, but quantal content (QC) was elevated >80% (*elav*-Gal4,24B-Gal4/+, 362.0±13.9 QC; *elav*-Gal4,24B-Gal4/42956, 677.6±29.9 QC; Fig. 6C). Thus, PMM2 specifically regulates evoked QC (Fig. 6C). These results indicate that the NMJ functional defect is not cell autonomous, because either pre- or post-synaptic PMM2 is sufficient to properly regulate neurotransmission strength. We therefore began to test non-cell-autonomous molecular mechanisms that could underlie the PMM2 function.

### PMM2 positively regulates the synaptic extracellular matrix proteinase pathway

Extracellular mechanisms in the highly glycosylated NMJ synaptomatrix provide an obvious answer to the PMM2 non-cell-autonomous phenotype. We were first guided to consider extracellular MMP pathways owing to the common tracheal break and melanization mutant phenotypes (Glasheen et al., 2010; Page-McCaw et al., 2007; Zhang and Ward, 2009) shared with PMM2 LOF (data not shown). Subsequently, recent work has shown that *mmp* mutants exhibit both NMJ structural and functional phenotypes that are strikingly similar to PMM2 LOF (Dear et al., 2016). Therefore, we examined the matrix metalloproteome at the NMJ, which includes secreted MMP1, glycosylphosphatidylinositol (GPI)-anchored MMP2 and their shared secreted tissue inhibitor of MMP (TIMP) (Dear et al., 2016; Kessenbrock et al., 2010; Page-McCaw et al., 2007). We hypothesized that PMM2-dependent glycan modification of these extracellular proteins, and/or their synaptic substrates, could provide a mechanism regulating NMJ structure and function.

**Fig. 7.**
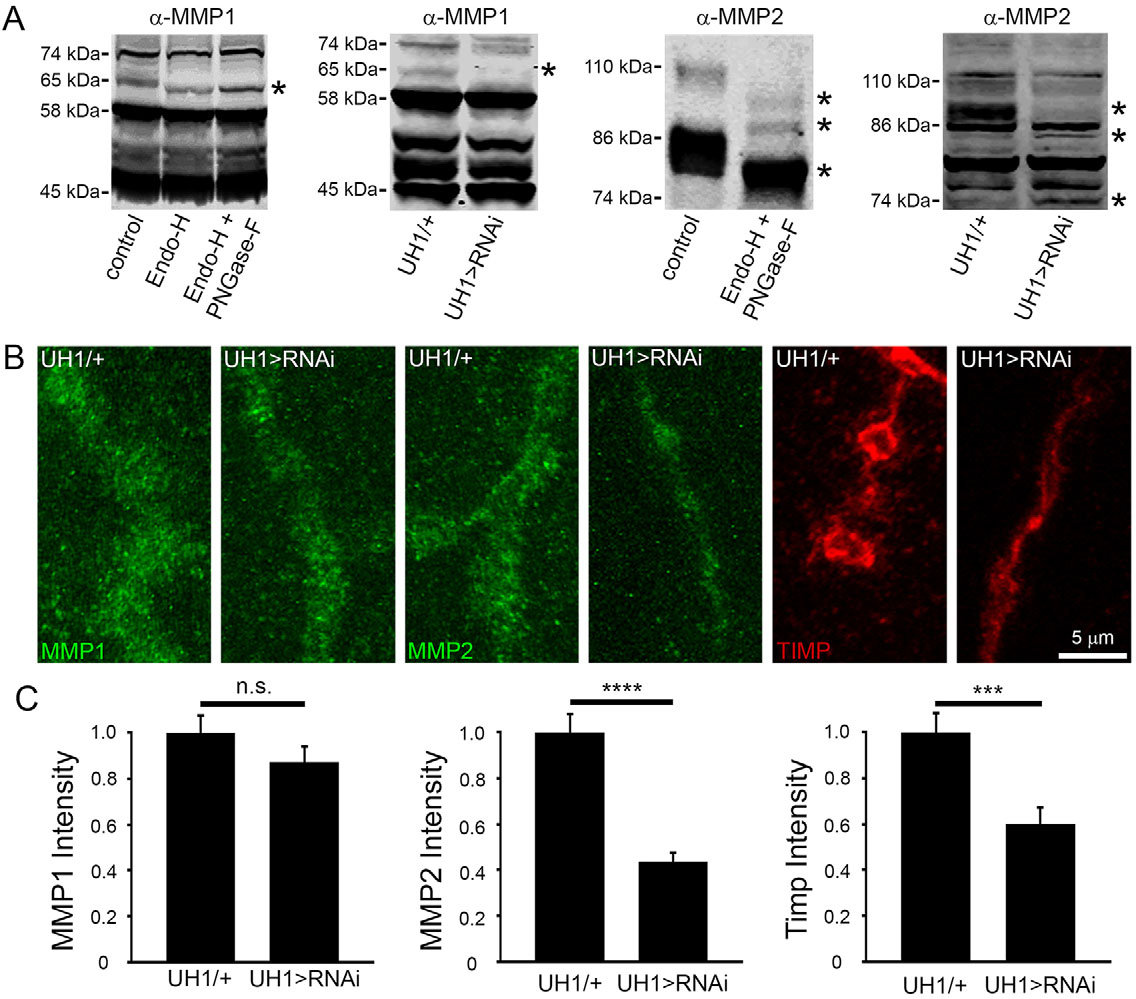
**Loss of PMM2 downregulates the synaptic matrix proteinase pathway.** (A) Representative western blots for MMP1 (left) and MMP2 (right); (1) with/without PNGaseF and EndoH enzymatic treatment to remove glycosylation, and (2) in UH1-Gal4/+ transgenic control and UH>RNAi^107619^ PMM2 knockdown conditions. The asterisks denote shifted bands. (B) Representative NMJ images for anti-MMP1 (green, left), anti-MMP2 (green, center) and anti-TIMP (red, right) in UH1-Gal4/+ controls and UH1-Gal4>RNAi^107619^. (C) Normalized quantification of fluorescent intensities for all three proteins. Significance: *P*≤0.001 (***), *P*≤0.0001 (****) and not significant (n.s.). Sample sizes: *n*≥20 NMJs/12 animals per genotype.

Consistent with this hypothesis, utilizing PNGaseF and Endoglycosidase-H (EndoH) to remove N-linked glycosylation (Song et al., 2011) revealed a clear reduction in MMP size (Fig. 7A), showing a high level of glycosylation (Glasheen et al., 2009; Jia et al., 2014). This change is much greater for membrane-anchored MMP2, in which all major isoforms show glycosylation-dependent shifts in size (Fig. 7A, asterisks), compared to secreted MMP1, in which only one minor isoform displays glycosylation. We next tested for non-glycosylated MMP forms in mutants using UH1-Gal4-driven *pmm2* knockdown (Fig. 7A). Loss of PMM2 results in multiple shifted (non-glycosylated) MMP bands compared to controls. Again, the effect is much greater for MMP2, with multiple bands showing a PMM2-dependent loss of glycosylation (Fig. 7A, asterisks). Consistently, NMJ labeling in *pmm2* RNAi compared to controls showed that the synaptic matrix metalloproteome is compromised, again particularly for MMP2 (Fig. 7B). PMM2 ubiquitous knockdown resulted in >50% reduction in MMP2 levels (UH1-Gal4>RNAi^107619^, 0.44±0.04, *n*=32) normalized to control (UH1-Gal4/+, 1.00±0.08, *n*=32; *P*<0.0001; Fig. 7C). In contrast, there was no significant change in MMP1 expression, although there was a slight decreasing trend (UH1-Gal4>RNAi^107619^, 0.87±0.07, *n*=20; UH1-Gal4/+, 1.00±0.07, *n*=24; Fig. 7B,C). TIMP expression was also reduced with PMM2 loss (UH1-Gal4>RNAi^107619^, 0.60±0.07, *n*=24) compared to control (UH1-Gal4/+, 1.00±0.08, *n*=24; *P*<0.001; Fig. 7B,C). These results show that synaptic MMP2 is strongly reduced by PMM2 removal, with a reduction also in synaptic TIMP levels. Given the importance of the synaptic metalloproteome in shaping Wnt trans-synaptic signaling (Dear et al., 2016), we next tested predicted PMM2 involvement.

### PMM2 positively regulates the Wg trans-synaptic signaling pathway

MMPs play an important role in Wg intercellular signaling by directly regulating the Wg co-receptor, the HSPG Dlp (Wang and Page-McCaw, 2014). Importantly, the same Wg-Dlp signaling pathway is a critical driver of structural and functional development at the *Drosophila* NMJ (Ataman et al., 2006, 2008; Kerr et al., 2014; Mathew et al., 2005) and is known to be modulated by glycan mechanisms (Dani et al., 2012; Jumbo-Lucioni et al., 2014; Parkinson et al., 2013). Wg binds the Frizzled-2 (Fz2) receptor, which is internalized and the C-terminus proteolytically cleaved (Fz2C) for transport via the Fz2C nuclear import (FNI) pathway (Ataman et al., 2006; Mathew et al., 2005) to modulate NMJ structure and/or function (Speese et al., 2012). Based on this extensive work, we hypothesized that PMM2 regulates MMP2-dependent Wg signaling to regulate NMJ structure and function, which underlies coordinated movement and maintained viability.

**Fig. 8.**
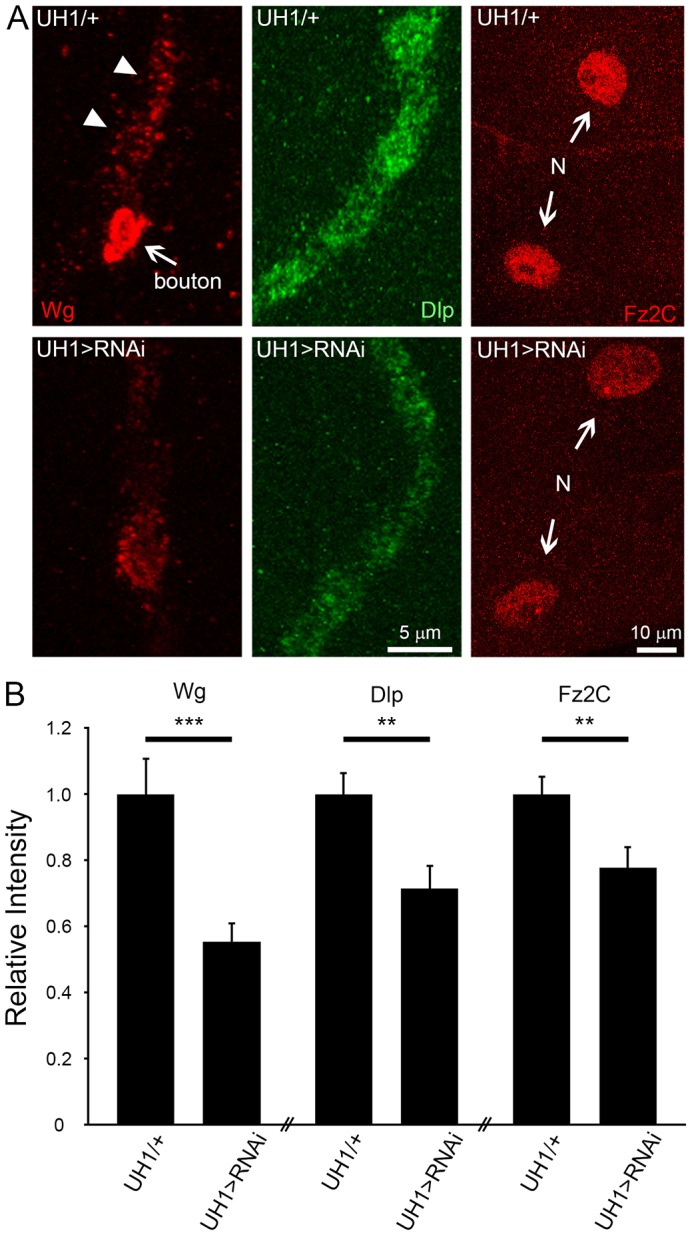
**Loss of PMM2 downregulates the trans-synaptic Wnt signaling pathway.** (A) Representative NMJ images for anti-Wg (red, left), anti-Dlp (green, middle) and anti-Fz2C (red, right) in UH1-Gal4/+ controls (top) and UH1-Gal4>RNAi^107619^ (bottom). Left: arrow indicates high Wg-expressing bouton, and arrowheads show low Wg-expressing boutons in control NMJ. Right: the arrows indicate two post-synaptic nuclei (N) in control and mutant muscle. (B) Normalized quantification of Wg, Dlp and Fz2C fluorescent labeling intensities. Significance: *P*≤0.01 (**) and *P*≤0.001 (***). Sample sizes: *n*≥24 NMJs or nuclei/12 animals per genotype.

All three components of the signaling pathway show clear downregulation with PMM2 loss (Fig. 8). Qualitative comparison of Wg ligand and Dlp co-receptor at the NMJ, and Fz2C cleavage and/or import into the post-synaptic muscle nuclei, all showed a reduction of pathway components and impairment of downstream signaling (Fig. 8A). Quantification of the extracellular Wg levels showed an ∼50% reduction with PMM2 ubiquitous knockdown (UH1-Gal4>RNAi^107619^, 0.55±0.05, *n*=32) normalized to control (UH1-Gal4/+ 1.00±0.11, *n*=30; *P*<0.001; Fig. 8B, left). Wg co-receptor Dlp is also reduced (UH1-Gal4>RNAi^107619^, 0.72±0.07, *n*=24) compared to control (UH1-Gal4/+, 1.00±0.06, *n*=24, *P*<0.01; Fig. 8B, middle). Finally, consequent Fz2C import into the muscle nucleus via the FNI pathway to mediate downstream signaling is significantly impaired with PMM2 ubiquitous knockdown (UH1-Gal4>RNAi^107619^, 0.78±0.05, *n*=24) compared to control (UH1-Gal4/+, 1.00±0.04, *n*=24), showing a significant decrease in trans-synaptic signaling (*P*<0.01; Fig. 8B). These results agree well with the recent report of strongly reduced Wnt signaling in the *Xenopus* CDG-Ia model (Himmelreich et al., 2015). We conclude that neurological impairments in the *Drosophila* CDG-Ia model similarly map to impaired Wnt signaling, leading to misregulated NMJ synaptogenesis and hence the underlying coordinated movement.

## DISCUSSION

We set forth to establish a *Drosophila* CDG-Ia (aka PMM2-CDG) genetic model through manipulation of the causative *PMM2* gene (Freeze et al., 2014). Using CRISPR-generated *pmm2*-null mutants and transgenic RNAi, we found that PMM2 levels correlated to coordinated movement abilities and lifespan (Fig. 1), as in individuals with CDG-Ia (Cylwik et al., 2013; Jaeken, 2013; de Lonlay et al., 2001). Humans with identical *PMM2* mutations present with a wide spectrum of movement defects (Marquardt and Denecke, 2003; Schneider et al., 2011), attributed to genetic and/or environmental factors that can be readily controlled in *Drosophila*. With tissue-specific drivers, we found a neural PMM2 impairment for coordinated movement (Fig. 1). Interestingly, weak neural knockdown of *pmm2* resulted in increased lifespan. Similarly, moderate impairments of oxidative-stress and dietary-restriction pathways have also been reported to extend lifespan (Mair et al., 2005; Min and Tatar, 2006; Ristow and Schmeisser, 2011). Like PMM2, severe impairments of these pathways result in reduced lifespan and early death, but more modest impairments extend lifespan via changes in metabolic rate, developmental conditioning and/or defense mechanisms. Null *pmm2* mutants displayed severe attenuation of glycoprotein glycosylation (Fig. 2), with reduced N-linked glycosylation diversity (Aoki et al., 2007). Similar global N-glycan losses occurred with strong *pmm2* RNAi throughout the larval neuromusculature, but not with weaker neural-targeted *pmm2* RNAi in the adult head (Fig. 2). Lipid-linked oligosaccharides (LLOs) used for protein attachment by OST activity are regulated at many levels (Gao et al., 2011). PMM2 loss should reduce LLO levels by inhibiting mannose-1-phosphate production and elevating the mannose-6-phosphate pool, which acts as a signal mediating LLO destruction, thereby increasing FOS levels (Fig. 3A; Gao et al., 2011). However, the increase in pauci-mannose structures with adult neuronal *pmm2* knockdown flies differentially alters glycan maturation. The mannose-6-phosphate increase might change the mannose phosphoisomerase (MPI) equilibrium, leading to interconversion of mannose-6-phosphate to fructose-6-phosphate to siphon LLO-toxic mannose-6-phosphate and mitigate LLO elimination.

Null *pmm2* mutants displayed elevated FOS levels (Fig. 3A). Increased phosphorylated FOS levels, predicted to be cleaved from LLO intermediates, likewise occur in CDG-Ia patient cells (Vleugels et al., 2011). Similarly, the zebrafish morpholino model shows increased FOS levels, with phenotype rescue via MPI co-reduction, suggesting causative mannose-6-phosphate elevation (Cline et al., 2012). Viable human CDG-Ia patients are typically heterozygous for *pmm2* mutations, resulting in partial loss of PMM2 (Matthijs et al., 1998; Monin et al., 2014). In *Drosophila*, partial LOF from neuron-targeted RNAi resulted in surprising resistance to glycosylation changes (Fig. 2C). Pauci-mannose glycans were increased, but high mannose and complex glycans unchanged. PMM2 regulates GDP-mannose availability for glycan production, but, following glycosylation by OST, further processing should be GDP-mannose independent (Cylwik et al., 2013). The increased pauci-mannose glycans resulting from PMM2 partial loss suggests that GDP-mannose levels influence glycan processing beyond the role as synthetic donors for mannosylation. Pauci-mannose glycan production is driven by the balance between Golgi exomannosidases, GlcNAc-transferase 1 and a hexosaminidase removing GlcNAc from nascent complex glycans (Stanley et al., 2009). Precursor abundances, including of donor and acceptor, influence expression and activity of glycan-processing enzymes. Reduced GDP-mannose likely skews the balance of enzyme activities that trim high-mannose to pauci-mannose, before they increase in complexity. Impacts on glycosylation independent of GDP-mannose were also evident by the observation of reduced NMJ lectin labeling with PMM2 loss (Fig. 3B). Importantly, both ECL and VVA bind mannose-free structures, most likely on O-linked glycoprotein backbones.

The nervous system is tightly regulated by glycans at multiple levels of development and function (Koles et al., 2007; Scott and Panin, 2014a,b; Seppo and Tiemeyer, 2000). NMJ architectural overelaboration and functional strengthening has been identified in many glycan mutants in our systematic genetic screens, including *sulf1*, *mgat1*, *galt* and *pgant* (Dani et al., 2012, 2014; Jumbo-Lucioni et al., 2014; Parkinson et al., 2013). Consistently, PMM2 loss showed striking NMJ overelaboration (Fig. 4), supporting the conclusion that glycans primarily inhibit synaptic morphogenesis. Null *pmm2* mutants already exhibited strong synaptic architecture defects within a day after hatching (Fig. 5), showing that PMM2-dependent glycan mechanisms brake the earliest stages of synaptic growth and differentiation. Glycosylation mechanisms also have key roles in modulating neurotransmission (Koles et al., 2007; Scott and Panin, 2014a,b), and PMM2 loss strongly increased NMJ function (Fig. 6). There was no effect on spontaneous synaptic vesicle release or post-synaptic amplitude, indicating a specific PMM2 role in limiting stimulus-evoked QC. Interestingly, neural- and muscle-targeted PMM2 knockdown had no effect on transmission; however, combined neural and muscle knockdown fully replicated the ubiquitous PMM2-loss phenotype (Fig. 6), indicating that the elevated transmission needs concomitant PMM2 removal both pre- and post-synaptically. One explanation is that semi-synaptic glycosylation might be sufficient to normalize transmission: loss of glycosylation from one side might be compensated for by the other side, because synapse transmission is highly regulated by both pre- and post-synaptic cells (Dani and Broadie, 2012). Another idea is that glycosylation could be provided by extracellular components from either synaptic partner cell.

The non-cell-autonomous defect occurring with PMM2 loss prompted us to investigate extracellular signaling mechanisms, which are tightly regulated by glycosylation at the NMJ (Dani et al., 2012, 2014; Parkinson et al., 2013; Jumbo-Lucioni et al., 2014; Rushton et al., 2012). In particular, MMPs play crucial roles shaping synapse structure and function (Dear et al., 2016; Kessenbrock et al., 2010; Page-McCaw et al., 2007; Sternlicht and Werb, 2001). PMM2 loss could impair MMP glycosylation or its ability to cleave improperly glycosylated substrates (Godenschwege et al., 2000; Llano et al., 2000, 2002; Pohar et al., 1999). PNGaseF and EndoH treatment to remove N-linked glycans showed that MMP2 isoforms are highly glycosylated, whereas only a tiny subset of MMP1 isoforms are glycosylated (Fig. 7A). GPI-anchored MMP2 also requires GDP-mannose, although GPI anchors require fewer donor mannose than N-linked glycans (de la Morena-Barrio et al., 2013). Importantly, *pmm2* RNAi similarly removed glycosylation from multiple MMP2 isoforms, with only a minor change to MMP1 (Fig. 7A). As predicted by PMM2-dependent glycosylation changes, MMP2 levels were strongly reduced at the NMJ synapse with *pmm2* RNAi, whereas those of MMP1 were not significantly altered (Fig. 7B,C). The TIMP regulator was also reduced in abundance with removal of PMM2, which would be predicted to help alleviate consequences of MMP2 loss, perhaps as a compensation mechanism (Dear et al., 2016). Importantly, recent work from our lab has shown that MMPs play crucial roles regulating NMJ structural and functional synaptogenesis via the control of HSPG receptors that modulate Wnt trans-synaptic signaling (Dear et al., 2016).

Recent work utilizing PMM2 morpholino knockdown in *Xenopus* revealed altered glycosylation of Wingless-type MMTV integration site family growth factor (Wnt) and reduction of Wnt signaling (Himmelreich et al., 2015). Similarly, we find that *pmm2* RNAi knockdown in *Drosophila* suppresses Wnt Wingless (Wg) signaling at the developing NMJ synapse (Fig. 8). With PMM2 loss, synaptic levels of Wg ligand and its HSPG co-receptor Dlp were both strongly reduced, and downstream signaling through the FNI (Speese et al., 2012) pathway was consistently downregulated (Fig. 8). These defects have been previously associated with the loss of synaptic MMP2, which acts via Dlp to regulate Wg trans-synaptic signaling to modulate both structural and functional NMJ development in the same direction (Dear et al., 2016). However, we have shown that PMM2 loss has myriad consequences on N-linked glycoprotein glycosylation, and therefore quite likely impacts NMJ synaptogenesis at multiple levels. Indeed, specifically targeted reduction in Wg signaling alone has previously been associated with decreased NMJ structural development and reduced function (Ataman et al., 2008; Kerr et al., 2014; Packard et al., 2002), which differs from the Wg attenuation associated with PMM2 loss. Therefore, PMM2 roles at the NMJ synapse likely reflect roles in multiple intersecting pathways that jointly control growth, structural differentiation and neurotransmission strength. Our future work will be aimed at deciphering other PMM2-dependent glycoprotein contributions, which combinatorially result in the structural and functional NMJ defects characterizing this CDG-Ia disease state model.

We hope that this new *Drosophila* model will prove instrumental for tackling the disease, and related CDGs, especially in regard to neurological symptoms (Grünewald, 2009; Jaeken, 2010). One avenue will be to dissect roles played by glycan precursors and mannose-6-phosphate buildup, by examining genetic interactions shifting the relative abundance of alternatively processed glycans and to alleviate increased FOS levels; for example, by using genetic MPI reduction or pharmaceutical MPI inhibitors in the benzoisothiazolone series (Sharma et al., 2011). One such agent, MLS0315771, has been shown to favor mannose-1-phosphate production in CDG-Ia patient fibroblasts and zebrafish embryos. Pharmacological tests in *Drosophila* could include assays to prolong lifespan, improve coordinated movement, and prevent NMJ structural and functional defects. The current standard of care for CDG-Ia patients is simply symptomatic treatment and disease management (Grünewald, 2009; Jaeken, 2013). The mouse model suggests beneficial dietary intervention, common for other metabolic disorders like classic galactosemia (Jumbo-Lucioni et al., 2014). However, mannose treatment has not been effective in restoring N-linked glycoprotein glycosylation levels in CDG-Ia patients (Thiel and Körner, 2013). Drug avenues to increase PMM2-dependent glycosylation are hypothesized, but there are no studies (Thiel and Körner, 2013). We expect that the relatively high speed of *Drosophila* disease model studies utilizing the powerful *Drosophila* genetic toolkit will open up new avenues for disease intervention. We propose here that targeting the matrix metalloproteome and Wnt signaling pathways offers potential new candidates to consider in developing future CDG-Ia treatments.

## MATERIALS AND METHODS

### *Drosophila* genetics

*Drosophila* stocks were grown on standard cornmeal/agar/molasses food in a 12 h light:dark cycle at 25°C. Mutants were generated with CRISPR/Cas9 (Gratz et al., 2013). Briefly, chiRNA targeting 5′-CATTGAAGCGTGATGAAATC-3′ and 3′-AGGATACGCAACGATTCTC-5′ sequences of *pmm2* were incorporated into a pU6-*Bbs*I-chiRNA plasmid (Addgene #45946). F1 progeny from *w^1118^* vas-Cas9 males [Bloomington *Drosophila* Stock Center (BDSC)# 51324] crossed to *w^1118^* Lig4 females (BDSC# 28877) were injected with both targeting plasmids (BestGene Inc., Chino Hills, CA). Injected animals were then crossed to a double balanced (TM3Sb/TM6Tb) mate. F1 males were then crossed to deficiency/balancer females [*w^1118^*; Df(3L)BSC380/TM6C, Sb^1^cu^1^ (BSCD #24404)] to identify lethal *pmm2* mutations. F1 males were recollected and mated with double balanced females to produce the *pmm2* mutant stocks. Mutant backcrossing and sequencing were performed with standard *Drosophila* genetic and PCR techniques. The *w^1118^* background stock was used as the control. RNAi studies were performed with neuronal-specific *elav*-Gal4, muscle-specific 24B-Gal4 and ubiquitous UH1-Gal4 transgenic drivers (Brand and Perrimon, 1993; Lin and Goodman, 1994; Rohrbough et al., 2007). Two *pmm2* UAS-RNAi lines, v107691 (Vienna *Drosophila* RNAi Center) and BDSC42956 (BDSC) were used, with *Gal4* drivers alone as transgenic controls.

### PCR methods

For reverse transcription quantitative PCR (RT-qPCR), total RNA was extracted using a Zymo Research Direct-zol RNA Miniprep Plus Kit with TRI reagent (R2070) with on-column DNase treatment. The Superscript VILO cDNA synthesis kit (11754-050) was used for cDNA synthesis. RT-qPCR was run on a Bio-Rad CFX96 with equal amounts of cDNA (2 ng for each trial). For expression quantification, the Pfaffl method was used with standards of known transcript number to quantify absolute cDNA number for target and reference genes. For the reference, ribosomal protein L32 (CG7939) levels were used for normalization of absolute cDNA quantity [(target/reference)×100]. The following primers were used for target and reference: *pmm2* forward 5′-AGGCTCGGATCTGGAGAAGA-3′, *pmm2* reverse 5′-AATGTCGTACTCGGCGAACA-3′; *L32* forward 5′-CGGTTACGGATCGAACAAGC-3′, *L32* reverse 5′-CTTGCGCTTCTTGGAGGAGA-3′. Samples and standards were run with gene-specific primers in duplicate trials. *n*=5 tissue collection samples and *n*=15 assay replicates. 50 first instars were used for RNA extraction.

### Behavioral assays

Egg lays were collected overnight on apple juice agar plates. Plates were then cleared of larvae, and newly hatched larvae collected after 1 h (*t*=0). Larval lifespan analyses involved daily counts. Adult lifespan analyses required two separate methods. For strong neural *elav*-Gal4>RNAi *pmm2* knockdown and *elav*-Gal4/+ controls, adults were collected at eclosion and maintained in laying pots with filter paper covering apple juice plates with yeast. For all other lifespan assays, adults were maintained in normal fly tubes on cornmeal/agar/molasses food. Adult survival was measured 3 times/week, with animals transferred to fresh plates or tubes. The comparative quantification of survival is reported as the time to which 50% of the animals remain viable [half-time survival (HTS)]. Adult and larval locomotion were assayed as previously described (Nichols et al., 2012; Sokolowski, 1980). Briefly, larvae were tested on apple juice agar plates with yeast paste spread around edges as an attractant. Individual larvae were placed in the middle of a plate and time-lapse-recorded under a dissection microscope with a Canon Rebel DSLR camera (Melville, NY). Locomotion was assayed as peristaltic waves per second (w/s) (Gjorgjieva et al., 2013). Adult motility was assayed by negative geotaxis and a ring locomotion assay. For geotaxis, adults were placed in empty fly vials for 15 min to acclimate, and then tubes were sharply tapped to put animals at the bottom (Nichols et al., 2012). Movement was recorded with a Canon Rebel DSLR camera and the percentage of animals to climb above 2 cm measured at timed intervals. To assay horizontal locomotion, a 4-cm circle was drawn in a large dish, and flies with amputated wings were placed in the middle of the circle. Movement was recorded with a Canon Rebel DSLR camera and the time required to traverse the circle measured.

### Glycomic analyses

Glycoproteins and free oligosaccharides (FOS) were prepared from staged collections of *pmm2*-null 1st instars or *elav*-Gal4>RNAi^42956^ adult heads by homogenization in aqueous/organic solvents and subsequent protein precipitation as described previously (Aoki et al., 2007). Briefly, the aqueous/organic homogenate was centrifuged with glycoproteins recovered in the pellet and FOS from the supernatant (Katoh et al., 2013). Precipitated proteins were washed with cold acetone and dried under a stream of nitrogen to produce samples stored desiccated at −20°C. Protein content was determined by bicinchoninic acid (BCA) assay of resolubilized material (Pierce). N-linked glycoprotein glycans were prepared from 1-mg aliquots by digestion with trypsin/chymotrypsin, followed by enzymatic release of glycans with PNGaseF (Aoki et al., 2007). FOS were separated by passage over a Sep-pak C_18_ cartridge column (Katoh et al., 2013). N-linked glycans and FOS were permethylated and analyzed by mass spectrometry (MS) using nanospray ionization coupled to linear iontrap and orbital Fourier transform mass analyzers (Discover NSI-LTQ/OrbitrapFT, Thermo-Fisher Scientific). MS spectra were collected over the range m/z=200-2000 and MS/MS fragmentation by collision-induced dissociation (CID; 30-40% normalized collision energy) was acquired over the same m/z range using the total ion mapping function of the XCalibur instrument software (version 2.0). Annotated glycans were validated by exact mass in full MS and by manual inspection of MS/MS spectra at each of the detected m/z values.

### Immunocytochemistry imaging

Immunocytochemistry studies were performed as described previously (Parkinson et al., 2013). Briefly, all animals were dissected, fixed and labeled identically in the same dish. Wandering 3rd or 1st instars were dissected in physiological saline containing 128 mM NaCl, 2 mM KCl, 4 mM MgCl_2_, 0.25 mM CaCl_2_, 70 mM sucrose, 5 mM trehalose and 5 mM HEPES (pH 7.1). Preparations were fixed in 4% paraformaldehyde for 10 min at room temperature (RT) in phosphate-buffered saline (PBS). Preparations were then either processed with detergent [PBS+1% bovine serum albumin (BSA)+0.2% Triton X-100] for intracellular labeling, or detergent-free (PBS with 1% BSA) for extracellular studies. Primary antibodies included: rabbit anti-horseradish peroxidase (HRP, 1:200; Sigma, St Louis, MO); conjugated CY2-, CY3- or CY5-HRP (1:250, Jackson Labs, West Grove, PA); mouse anti-Wingless [Wg, 1:2; Developmental Studies Hybridoma Bank (DSHB), University of Iowa, Iowa City, Iowa]; mouse anti-Discs-large (DLG, 1:200; DSHB). Lectins included: *Vicia villosa* agglutinin (VVA-Tritc, 1:200; E.Y. Laboratories, San Mateo, CA) and *Erythrina cristagalli* lectin (ECL-biotin, 1:250; Vector Labs). Secondary Alexa fluorophore antibodies (Invitrogen, Grand Island, NY) included: goat anti-mouse 488 and 568 (1:250), goat anti-rabbit 488 and 568 (1:250), and streptavidin 488 and 594 (1:250). Primary antibodies and lectins were incubated at 4°C overnight; secondary antibodies were incubated at RT for 2 h. Samples were mounted in Fluoromount-G (Electron Microscopy Sciences, Hatfield, PA). All preparations were imaged using identical parameters. *Z*-stacks were taken with a Zeiss LSM510 META laser-scanning confocal using a 63× Plan Apo oil-immersion objective. Optical sections were imaged starting above and ending below the NMJ or muscle nuclei to encompass their entirety. Stacks were projected on the *Z*-axis for maximum intensity, with NMJ or nuclei signals highlighted and average intensity quantified using ImageJ (Abramoff et al., 2001).

### Western blot analyses

Tissues were homogenized in buffer (1% SDS, 50 mM Tris-HCl, 150 mM NaCl) with protease inhibitors, heated (70°C, 10 min) and centrifuged (16,100 ***g***, 10 min). Supernatant was split into two tubes (+/− enzyme) and glycosidase treatment done following the manufacturer’s instructions [New England Biolabs (NEB), Ipswich, MA]. Briefly, samples in denaturing buffer (NEB) were heated (10 min, at 95°C) then cooled to RT. Denatured samples were treated with or without (buffer alone) EndoH (NEB) and PNGase-F (NEB) at 1 µl enzyme/20 µg protein in 1× G5 buffer (NEB) overnight at 37°C. Samples were assayed with western blot SDS-PAGE using 10% Bis-Tris gels and western blot analysis. Membranes were blocked in 2% milk in tris-buffered saline (TBS) for 1 h at RT. Mmp antibodies (1:1500) were incubated overnight at 4°C, then washed for 5 min (×6) in TBS+0.1% Tween-20 (TBST). Goat secondaries (1:10,000; Rockland, Limerick, PA) were incubated for 1 h at RT. Blots washed for 5 mins (×6) in TBS-T were imaged using an Odyssey Infrared Imaging System.

### Electrophysiology

Excitatory junctional current (EJC) recordings made using two-electrode voltage-clamp (TEVC) were done as previously reported (Parkinson et al., 2013). Briefly, wandering 3rd instars were glued with 3M Vetbond adhesive (World Precision Instruments, Sarasota, FL) to sylgard-coated glass coverslips, cut longitudinally along the dorsal midline, internal organs removed and sides affixed down for neuromusculature access. Peripheral nerves were cut at the ventral nerve cord. Recordings were done at 18°C in saline consisting of 128 mM NaCl, 2 mM KCl, 4 mM MgCl_2_, 1 mM CaCl_2_, 70 mM sucrose, 5 mM trehalose and 5 mM HEPES (pH 7.1), imaged using a Zeiss Axioskop microscope with 40× immersion objective. A fire-polished glass suction electrode was used for evoked nerve stimulation with 0.5 ms suprathreshold stimuli at 0.2 Hz from a Grass S88 stimulator (Rohrbough et al., 2007). Muscle 6 in abdominal segments 2/3 was impaled with two microelectrodes of 10-15 MΩ resistance filled with 3 M KCl, and clamped (−60 mV) using an Axoclamp-2B amplifier (Molecular Devices, Sunnyvale, CA). EJC records were filtered at 2 kHz. To quantify EJC amplitudes, ten consecutive traces were averaged. Spontaneous miniature EJC (mEJC) records were made in 2-min sessions and filtered at 200 Hz with a low-pass Gaussian filter prior to quantification. Clampex software was used for all data acquisition, and Clampfit software for all data analyses (Molecular Devices, Sunnyvale, CA).

### Statistics

All statistical analyses were performed using GraphPad InStat3 software (La Jolla, CA). Student's *t*-tests were used for pairwise comparisons, and ANOVA with appropriate post-hoc testing was used for all data sets of three or more comparisons. Nonparametric methods were used for data sets lacking normal distribution. Fisher's exact tests were used to analyze contingency tables for adult behavioral data. Data are shown as mean±s.e.m. in all figures, with significance presented as *P*≤0.05 (*), *P*≤0.01 (**), *P*≤0.001 (***) and *P*≤0.0001 (****).
